# *Notes from the Field:* Public Health Efforts to Mitigate COVID-19 Transmission During the April 7, 2020, Election ― City of Milwaukee, Wisconsin, March 13–May 5, 2020

**DOI:** 10.15585/mmwr.mm6930a4

**Published:** 2020-07-31

**Authors:** Heather Paradis, Julie Katrichis, Michael Stevenson, Nicholas Tomaro, Rachel Mukai, Griselle Torres, Sanjib Bhattacharyya, Jeanette Kowalik, Karen Schlanger, Eva Leidman

**Affiliations:** ^1^City of Milwaukee Health Department, Wisconsin; ^2^CDC COVID-19 Response Team.

Wisconsin was the first state to hold an election with in-person voting after stay-at-home orders were issued to limit transmission of SARS-CoV-2, the virus that causes coronavirus disease 2019 (COVID-19). The statewide primary election, held on April 7, 2020, occurred fewer than 2 weeks after the statewide “Safer at Home” order* became effective on March 25.

On March 3, 2020, CDC published interim guidance to prevent spread of SARS-CoV-2 at polling locations (*1*). Mitigation measures in line with the CDC guidance and additional measures were implemented in the city of Milwaukee (in Milwaukee County) to prevent the transmission of SARS-CoV-2 at in-person polling venues (Supplementary Table, https://stacks.cdc.gov/view/cdc/90768). In addition to the nearly 500 poll workers, election inspectors, and chief inspectors, Milwaukee city health department personnel and the Wisconsin National Guard were assigned to support mitigation efforts at each of five Milwaukee polling sites and the central count location. Mitigation measures implemented at the direction of the city health department complemented public messaging campaigns to encourage absentee voting. According to the Milwaukee Election Commission, comparing the number of persons voting in the spring of 2016 with those voting in the spring of 2020, the percentage of persons who voted by absentee mail-in ballots increased approximately fifteenfold, from 4.1% (6,874) to 68.0% (64,750) of voters; those who voted early (either in person or curbside [i.e., voting while remaining in their vehicle or at the voting place entrance]) increased by 160%, from 4.7% (7,949) to 12.2% (11,612). Although the proportion of those who voted in person on election day decreased 78%, from 91.2% (153,458) to 19.8% (18,806),^†^ local news media reported long waiting times at Milwaukee voting locations on election day.^§^ Overall, the number of persons who voted decreased 43%, from 168,281 to 95,168, and the number of polling sites decreased from 181 to five.

Laboratory-confirmed COVID-19 cases and epidemiologic data were used to characterize SARS-CoV-2 transmission from March 13, when the first case was confirmed in Milwaukee, through May 5, or 4 weeks following the election. Case counts, hospitalizations, and exposure data (including voting method ascertained using a standardized voting module) were obtained from the Wisconsin Electronic Disease Surveillance System (WEDSS).^¶^ Cases were reported by date of specimen collection or report if unavailable. Fatality data were obtained from the Milwaukee County Medical Examiner.

An estimated 95% of persons with COVID-19 develop symptoms within 2–14 days after exposure (*2*–*4*); therefore, persons infected at polls would be expected to develop symptoms during April 9–21. Among 2,789 COVID-19 cases, 642 related hospitalizations, and 137 COVID-19–associated deaths reported during March 13–May 5, 572 (21%) cases were reported during this expected incubation period (i.e., April 9–21) (Figure), compared with 693 (28%) cases reported during the 13 days preceding this incubation period (i.e., March 27–April 8). Among the 572 cases reported during April 9–21, 316 (55.2%) patients did not report their voting status, and 219 (38.3%) did not vote; 37 (6.5%) reported voting. Among these 37 COVID-19 patients who voted, 17 (45.9%) reported voting using an absentee mail-in ballot, 14 (37.8%) voted in person, and six (16.2%) voted curbside. During April 17–26 (the estimated interquartile range of the interval from illness onset to death for a person infected on election day), 24 deaths were reported, 33% fewer than the 36 deaths reported during the preceding 10 days (April 7–16) (Figure) (*5*). After a peak in hospitalizations during the last week in March, hospitalizations gradually declined.

**FIGURE:**
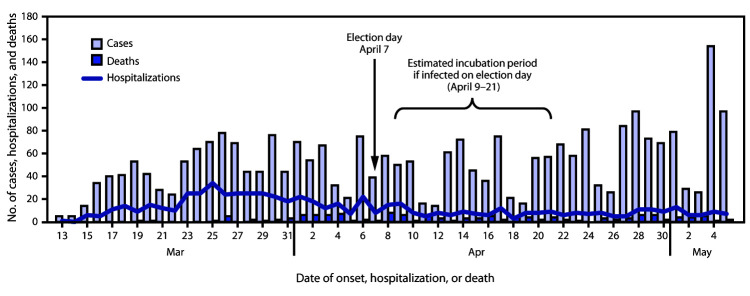
Number of reported COVID-19 cases, hospitalizations, and associated deaths — Milwaukee, Wisconsin, March 13–May 5, 2020* **Abbreviation:** COVID-19 = coronavirus disease 2019. * Based on available evidence, for a person exposed to SARS-CoV-2 on election day, the estimated incubation period (2–14 days) was April 9–21; the estimated median interval from illness onset to death was estimated to be 10 days (corresponding with April 21).

These data provide an initial assessment of potential impacts of public health efforts to mitigate COVID-19 transmission during an election. No clear increase in cases, hospitalizations, or deaths was observed after the election, suggesting possible benefit of the mitigation strategies, which limited in-person voting and aimed to ensure safety of the polling sites open on election day. Epidemiologic trends were likely also influenced by a relatively lower turnout of voters overall compared to spring 2016.

These data provide preliminary evidence that CDC’s interim guidance for ensuring various voting options, encouraging physical distancing, personal prevention practices, and employing environmental cleaning and disinfection lower COVID-19 transmission risk during elections (*1*). Further risk reduction can be achieved by fully implementing CDC interim guidance, which recommends longer voting periods, and other options such as increasing the number of polling locations to reduce the number of voters who congregate indoors in polling locations.

## Footnotes

* https://content.govdelivery.com/attachments/WIGOV/2020/03/24/file_attachments/1409408/Health%20Order%20%2312%20Safer%20At%20Home.pdf.

† https://elections.wi.gov/clerks/svrs.

§ https://www.jsonline.com/story/news/politics/elections/2020/04/07/wisconsin-election-milwaukee-voters-brave-long-wait-lines-polls/2962228001/.

¶ https://www.dhs.wisconsin.gov/wiphin/wedss.htm.
